# Correspondence between Dr. C. J. B. Williams and a Homœopathic Practitioner

**Published:** 1850-07

**Authors:** 


CORRESPONDENCE BETWEEN DR. C. J. B. WILLIAMS AND A HOMŒOPATHIC PRACTITIONER.

The *London Lancet* of March 23d, contains the following correspondence.

*The Letter.*

" —— Street, Friday, 22d February, 1850.

" DEAR SIR,—I am very desirous of having your opinion in a case of suspected disease of the heart. The patient is the Hon. Mrs. ——, at present residing with Lady ——, —— Square. Will you have the goodness to inform me at what hour on Monday it would be convenient for you to see Mrs —— ?

" I think it right to state that Mrs. —— has been for many years a convert to homœopathy, and that I, as you possibly may have heard, practise that system of treatment. I mention this, because you may have some objection to meet a homœopathic physician in consultation, and I should much regret if I were the means of inducing you to do anything distasteful to you in ignorance of the above facts. I may however, mention that it is as a matter of diagnosis rather than of treatment that your opinion is desired, and that my friends, Sir — —— and Dr. ——, have seen the case with me on former occasions. I remain, dear Sir, your very obedient servant, " ——."

" To Charles J. B. Williams, Esq. M. D. etc."

*The Reply.*

" 7 Holles Street, Cavendish Square, 23d, 1850.

" DEAR SIR,—I am obliged to you for your courtesy in wishing to have my opinion on the diagnosis of the case of the Hon. Mrs. ——, and for your candor in apprising me that she is under homœopathic treatment; but under these circumstances I must beg you to excuse my attendance.

" Believing, as I firmly do, that the so-called 'homœopathic system' is an entire fallacy, and therefore calculated to do much injury to those on whom it is practised, I consider it *to be my duty to do nothing that can, directly or indirectly, countenance or aid it;* and it appears to me, that to meet a homœopathic physician in consultation, and to assist in the diagnosis of a case professedly under homœopathic treatment, would have such an effect.

" I need scarcely add, that I have no personal feelings in the matter. And hoping that you will soon return to the legitimate domain of rational medicine, " I remain, dear Sir, yours faithfully,

" To Dr. ——. " C. J. B. WILLIAMS.

Dr. Williams' conduct in this case is most honorable and worthy of imitation: but the adoption of any other course by a leading physician, would have been a heavy blow and a great discouragement to the practitioners of legitimate medicine. Minor men may, (with their guinea bribes) be allowed to dally with 'entire fallacies,' but the consulting physician, who meets a homeopath, a hydropath, a chrons-thermalist, or any charlatan, must be carefully repudiated by the profession. We wish this hint to be acted upon: a laxity prevails.

